# The Dynamics of the ESCRT Machinery in Open Mitosis from Physiology to Pathology

**DOI:** 10.3390/cells14211681

**Published:** 2025-10-27

**Authors:** Mattia La Torre, Federica Cannistrà, Romina Burla, Isabella Saggio

**Affiliations:** 1 Department of Biology and Biotechnologies “Charles Darwin”, Sapienza University, 00185 Rome, Italy; 2 CNR Institute of Molecular Biology and Pathology, 00185 Rome, Italy

**Keywords:** nuclear membrane, cell division, nucleopathies, cancer, cell migration, neurodegeneration

## Abstract

**Highlights:**

**What are the main findings?**

1. In open mitosis, ESCRTs type III are recruited to the anaphase/telophase chromatin core by BAF1 and LEM2; CHMP7 and IST1 bring in VPS4 and Spastin to couple nuclear envelope reformation with spindle microtubule disassembly. Canonical ESCRTs type I and II have not yet been identified as part of this nuclear envelope ESCRT module.
2. Beyond mitosis, the same axis operates during interphase nuclear envelope rupture and repair. ESCRT factors also contribute to nuclear pore complex quality control.

**What are the implication of the main findings?**

3. Correct ESCRTs type III assembly in mitosis is essential for nuclear reformation and genome integrity. ESCRT dysfunction causes DNA damage, spindle clearance defects, and chromosomal instability, mechanistically linking ESCRTs to disease including cancer and neurodegeneration.
4. ESCRT-dependent nuclear envelope surveillance pathways are emerging as therapeutic entries. Moreover, candidate new factors, such as the ESCRT type I like factor AKTIP, suggest additional druggable nodes.

**Abstract:**

The Endosomal Sorting Complex Required for Transport (ESCRT) is a highly conserved machinery best known for its role in endosomal trafficking and membrane remodeling. Increasing evidence shows that ESCRT components are also key regulators during open mitosis, where precise membrane dynamics are essential for nuclear envelope reformation and spindle disassembly. In this review, we explore how the ESCRT machinery coordinates mitotic processes under physiological conditions and how their dysregulation contributes to genomic instability, altered cell division, and disease. We highlight recent findings on the spatiotemporal control of ESCRT recruitment at mitotic membranes, the interplay with chromatin and nuclear envelope-associated factors, and the consequences of defective ESCRT function in pathological contexts such as cancer and neurodegeneration. By connecting molecular mechanisms with cellular outcomes, we provide an integrated view of how the ESCRT machinery acts as critical guardian of mitotic fidelity and offer some routes for the identification of potential therapeutic targets in human disease.

## 1. The Organization of the ESCRT Machinery

The ESCRT machinery is multi-complex, working at several sites and involved in different cellular processes. These include the intracellular trafficking and, more specifically, the formation of multivesicular bodies [1], the final phase of cell division [2], that requires the physical separation of the daughter cells, and the repair of nuclear envelopes, which happens post-mitotically and in interphase ruptured nuclei [3,4]. The ESCRT factors composing the machinery are conserved through evolution. The first studies describing ESCRTs started in the year 2000 in eukaryotes, prevalently in yeast, followed by studies in mammals. Recent analyses highlight ESCRT activity in procaryotes too [5,6].

**Figure 1:**
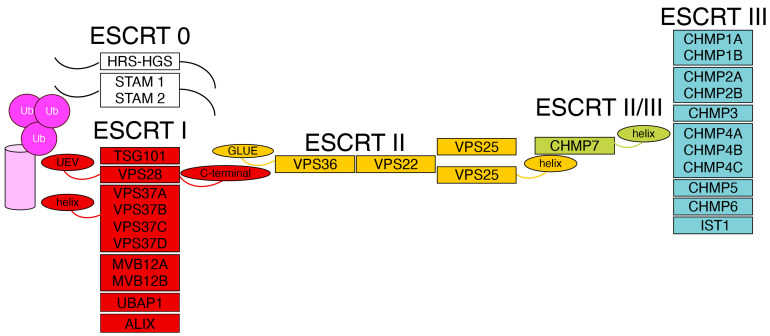
The organization of the ESCRT machinery Schematic representation of the ESCRT complexes. The N-terminal regions of the ESCRTs type 0, HRS-HGS and STAM, are involved in the recognition of ubiquitinated cargo proteins (magenta) and in the recruitment of the ESCRT I complex (red). The winged-helix domain of the ESCRT I subunit VPS37 and the UEV domain of TSG101 interact with ubiquitinated cargo. ESCRTs type I recruit ESCRTs type II (yellow) through an interaction between the C-terminal region of the ESCRT type I VPS28 and the GLUE domain of the ESCRT type II VPS36. ESCRTs type II assemble into a Y-shaped structure and, via the winged-helix domain of VPS25, recruit the ESCRT subunit CHMP7 (light green). CHMP7, in turn, recruits ESCRTs type III (light blue) through its winged-helix domain.

The ESCRT factors are regrouped into four types, from 0 to IV. The type 0 and type I ESCRT factors define, in principle, the site of action of the machinery (Figure 1). ESCRTs type II are a bridge to the recruitment of ESCRTs type III or late ESCRT factors. The latter are needed for the action of the machinery; namely, they are directly involved in membrane remodeling and scission. In human cells, the main components of the ESCRT machinery are as follows: type 0—HRS-HGS, STAM1 and STAM2. ESCRTs; type I—TSG101, VPS28, VPS37, in its four isoforms VPS37 A to D, MVB12 A and B, UBAP1 and Alix; type II—VPS25, VPS36 and VPS22; and ESCRTs type III—CHMP1A and B, CHMP2A and B, CHMP3, CHMP4A to C, CHMP5, CHMP6, CHMP7, and IST1 (or CHMP8). Physical and in silico prediction studies have defined a rod shaped hetero-tetrameric structure for ESCRTs type I, and a Y-shaped structure for ESCRTs type II [7,8,9]. Single ESCRT III factors are small proteins containing five to six alpha helices that form polymers and have a dynamic structural behavior. Indeed, they are detected as soluble factors, or as filaments [10,11] and, on membranes, they can typically spiralize [12]. 

VPS4 is an ATPase, which is associated with the ESCRT machinery. VPS4 is recruited by ESCRTs type III via an interaction between MIT (Microtubule-Interacting and Trafficking) and MIM (MIT Interacting Motifs) domains [13]. VPS4 has a pivotal role in the control of the machinery: it regulates the disassembly and recycling of the ESCRT type III filaments [14,15]. The factor CC2D1B is also associated with the ESCRT machinery and controls the accumulation of ESCRTs type III during nuclear envelope reformation and repair [16]. 

## 2. The Function of the ESCRT Machinery Across Species

To interpret the function of the ESCRT machinery, it is relevant to analyze its components through an evolutionary lens. Indeed, this perspective highlights multiple concepts which help in interpretating the roles played by this machinery. This applies both to the known functions and to those which are currently only being hypothesized. The first element that strikes, when comparatively analyzing the genes encoding the components of the ESCRT machinery in different species, is their conservation. Indeed, ESCRT factors’ encoding genes are retrieved from Archaea to mammals, from Escherichia coli to Drosophila melanogaster [17,18]. The second aspect emerging from the evolutionary analysis is that the ESCRT machinery-associated ATPase VPS4 is present in most of the organisms, as much as at least one representative of the ESCRTs type III. On the other hand, this is not the case for ESCRTs type I and II. Indeed, for example, ESCRTs type I are abundant in humans, but can be absent in less complex organisms [13,19,20]. This evolutionary conservation of the ESCRTs type III core components and of the ATPases indicates a pivotal role for the machinery in the life of a cell, and, on the other end, they suggest common mechanisms of action for ESCRTs type III and VSP4 across the different species (Table 1).

**Figure 2:**
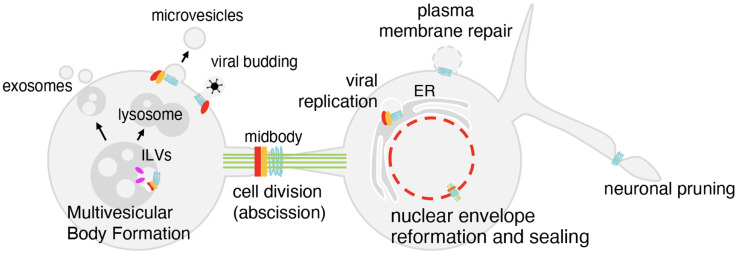
The functions of the ESCRT machinery. At endosomes, the ESCRT machinery controls intraluminal vesicle (ILV) formation, leading either to lysosomal degradation or exosome release. During cytokinesis, ESCRTs type I (red), II (orange) and III (light blue) are recruited to the midbody to finalize abscission. At the nuclear envelope, ESCRTs type III cooperate to seal membranes after mitosis and repair ruptures. ESCRTs also act at the plasma membrane to close lesions, at the neuronal branches to drive pruning, and are hijacked by viruses to promote budding.

The current knowledge on ESCRT biology in humans originates from studies in yeast. Indeed, from yeast to humans, the main functions of the ESCRT machinery are conserved. One such function is the control of multivesicular bodies formation, which regulate the recycling of cellular receptors. Indeed, in this process, receptors are ubiquitinated and endocytosed from the plasma membrane; at this stage they interact with early ESCRT factors and are internalized as endosomal intraluminal vesicles [1]. These multivesicular bodies are successively fused with lysosomes. The destiny of receptors can be their degradation or their release as exosome components. If the first route influences the cell in an autocrine manner, exosomes exert a paracrine, cell-to-cell communication effect [21] (Figure 2). 

**Table 1:** ESCRT proteins in humans and in model organisms. ESCRT proteins show a high degree of conservation. The table reports ESCRT protein names in yeast (*S. cerevisiae* and *S. pombe*), fly, and human. Alternative ESCRT protein names, when present, are given in parentheses; - indicates that no ortholog has been identified.

	*S. cerevisiae*	*S. pombe*	*D. melanogaster*	*H. sapiens*
ESCRT 0	Vps27 [22]	Vps27 (Sst4) [23]	Hrs [24]	HRS (HGS) [25]
	Hse1 [22]	Hse1 [23]	Stam [26]	STAM 1 [27]
				STAM2 [27]
ESCRT I	Vps23 (Stp22) [7]	Sst6 [28]	Erupted (Tsg101) [29]	TSG101 [30]
	Vps28 [7]	Vps28 [23]	Vps28 [31,32]	VPS28 [33]
	Vps37/Srn2 [7]	-	Vps37a [34,35]	VPS37A [36]
			Vps37b [34,35]	VPS37B [36]
				VPS37C [37]
				VPS37D [36]
	Mvb12 [7]	-	Mvb12 [38]	MVB12A [39,40]
				MVB12B [39,40]
ESCRT II	Vps36 [9]	Vps36 [28]	Vps36 [41,42]	VPS36 (EAP45) [8,43,44]
	Snf8 (Vps22) [9]	Dot2 [45]	Lsn/Vps22 [31,46]	VPS22 (EAP30) [43]
	Vps25 [9]	Vps25 [47]	Vps25 [31,48]	VPS25 (EAP20) [43]
ESCRT III	Vps2 (Did4) [49]	Vps2 (Did4) [47]	Vps2 [50]	CHMP2A [49]
				CHMP2B [49]
	Vps24 (Did3) [49]	Vps24 [28,51]	Vps24 [31]	CHMP3 [49]
	Snf7 (Did1, Vps32) [49]	Vps32 [28,51]	Shrb [31,52]	CHMP4A [11]
				CHMP4B [11]
				CHMP4C [11]
	Vps60 [49]	Vps60 [53]	Vps60 [54,55]	CHMP5 [55]
	Vps20 [49]	Vps20 [23]	Vps20 [56]	CHMP6 [57]
	Did2 (Vps46) [49]	Did2 [58]	Chmp1 [55]	CHMP1A [59,60]
				CHMP1B [59,60]
	Chm7 [61,62]	Cmp7 [58,63]	CG5498 *	CHMP7 [64,65,66]
	Ist1 [67]	Ist1 [58,68]	Ist1 [54,55]	IST1 (CHMP8) [69]
ESCRT related protein	Vps4 [70]	Vps4 [51]	Vps4 [71,72]	VPS4A [15,73] VPS4B [15,73]

* predicted ortholog (Flybase).

A second key biological process regulated by the ESCRT machinery and conserved through evolution is cell division (Figure 2). Indeed, simple organisms such as Archaea and the Red alga share with humans the implication of the ESCRT machinery in this process [74,75,76]. Here, during the final stages of cytokinesis, in the abscission phase, ESCRTs type I, II, and III are recruited at the midbody, which is the central region of the 1um wide microtubule-rich intercellular bridge linking the daughter cells [2]. In humans, ESCRTs type I and II are recruited to the midbody by CEP55 [77]. In the late abscission stage, ESCRTs type III, together with the microtubule severing enzyme Spastin, finalize the disassembly of the intercellular bridge completing the separation of the daughter cells [2,60]. This process is prototypical of the ESCRT machinery; it contributes to cell division of unicellular organisms and also has a potential significant role in determining the characteristics of the daughter cells in multicellular organisms, including their differentiative properties, their stemness potential, and their senescence [78,79].

Beyond multivesicular body formation and abscission, the ESCRT machinery is involved in many other functions (Figure 2). It contributes, for example, to neuronal pruning, impacting organismal development [71]. 

The ESCRT machinery is also exploited by invading organisms [80]. The need for the ESCRT machinery has been described, for example, for the budding of HIV from the cell [33], or for the egress of EBV particles from the nucleus during infection [81]. Studies based on ESCRT–virus interaction have highlighted the pivotal role of short peptides as, for example, that of the PTAP motif of the HIV1 protein GAG which interacts with the ESCRT type I TSG101 [82,83]. It has also been suggested that the GAG protein interacts with type III ESCRTs, including CHMP2, CHMP4, and CHMP6 [84,85].

If ESCRTs are hijacked by invading microorganisms, the machinery also plays a role in protecting the cell from invasion. Indeed, the machinery can, for example, repair cell membrane ruptures caused by bacteria [86,87].

Finally, the ESCRT machinery plays a critical role at the nuclear envelope, mitotically and in interphase nuclei [3,4], as detailed in the following sections.

## 3. The Process of Open Mitosis

Mitosis is a complex phenomenon crucial for eukaryotic cells. Indeed, during cell division, the mother cell not only must ensure the distribution of cytoplasmic material, but, importantly, must also divide the genetic material equally and correctly into the daughter cells. This process is made even more delicate by the necessity to guarantee in the newly formed cells the separation of the genetic material between nucleus and cytoplasm. 

Mitosis has been cytologically known since the 19th century, and its complexity has made it an object of intense study, which has involved different disciplines in an increasingly interconnected manner, from physics to biochemistry, from high-resolution microscopy to structural biology. Importantly, if the bases of mitosis were described already by W. Flemming long ago, there are still protein complexes and biological processes yet to be defined, or redefined [88]. 

Mitosis is typically divided into the prophase, metaphase, anaphase, and telophase. In eukaryotic cells, two principal modes of mitosis have evolved: open and closed [89]. In open mitosis, typical of multicellular organisms, the nuclear envelope breaks down before chromosome segregation, allowing the mitotic spindle to assemble in the cytoplasm and capture chromosomes directly. The nuclear envelope then re-forms around the two sets of separated chromatids at the end of division [90]. In contrast, closed mitosis, which is characteristic of many lower eukaryotes such as fungi, occurs entirely within an intact nucleus. Here, spindle fibers form inside the nuclear compartment, and chromosome segregation takes place without nuclear envelope disassembly [91]. From an evolutionary perspective, closed mitosis is considered the more ancient mechanism, while open mitosis appears to have been independently modified several times during evolution [89]. 

Of course, there are numerous reviews dedicated to mitosis, and this is not the scope of this paper, but we want to highlight some elements of open mitosis which are instrumental to the analysis of the function exerted by the ESCRT machinery in this process.

In open mitosis (from now on mitosis), prophase and metaphase are cytologically recognizable by the observation of microtubule spindle assembly and by the compaction of chromatin/DNA into chromosomes/chromatids. Anaphase and telophase are recognized by the observation of the separation of chromatids and by the reassembly of the nuclear envelope as a continuous membrane around the progressively reorganizing chromatin [90,92].

The starting of mitosis, the prophase, coincides with the organization of chromatin into compact chromosomes. Human chromosomes vary significantly in length. The largest chromosome, Chromosome 1, indeed has a length of approximately 85 mm when unfolded (249 million base pairs), while the smallest, Chromosome 21, is only 16 mm (48 million base pairs). In this case, it would be better to specify the range of chromosome lengths rather than the average value (48 mm). To reach the condensed format, the extended DNA fiber is wrapped around nucleosomes and extensively folded. The topological organization of mitotic chromosome loops has been experimentally demonstrated and simulated with polymers. It has been shown that chromosome compaction requires condensin, a ring-shaped protein complex, which has a key role in promoting the formation of chromosome bodies as also suggested by the fact that it is able to induce chromosome condensation even in the absence of nucleosomes [93]. Beyond condensin, the protein Ki-67, contributes to the structural organization of chromosomes. This aspect has been related to the surfactant properties of Ki-67, which indicate that this factor can act as a biological moiety for the dispersion of mitotic chromosomes. This biological function is supported also by cytological data which have highlighted Ki-67 distribution in brush-like structures around chromosomes [94].

If chromosome compaction and individualization is a determinant of mitotic prophase and a critical element for their correct distribution in the daughter cells, the dynamic organization of the spindle microtubules represents a second central aspect in the starting of the mitotic process. The spindle was first described in plant cells by Eduard Strasburger, who also coined the term spindle apparatus, and in animal cells by Oscar Hertwig and Eduard van Beneden in 1875 and 1876 [95,96]. Flemming, in 1882, introduced the term mitosis and provided the first detailed description of the spindle [97]. Since then, many studies have highlighted its role, composition, and dynamic nature. Microtubules are dimers of alpha- and beta-tubulin assembled into a polar protofilament with minus and plus ends. Nucleation of spindle microtubules, in all human cells but in meiotic cells, originates from centrosomes [98]. During metaphase, chromosomes attach to the spindle fibers to become organized on the metaphase plate. The interaction happens between the kinetochores, multi-protein structures present on each chromatid, attached to specialized chromatin, and the microtubules. Kinetochores contain the motor enzymes which represent essential elements for microtubule and chromosome dynamics. These include dyneins and kinesins [99]. By a search-and-capture process along with dynamic rearrangements, chromosomes position at microtubule plus ends. When all kinetochores are properly attached, a cell cycle checkpoint—the spindle checkpoint—is released and the mitotic process proceeds further into anaphase [100]. While chromosomes are distributed at the spindle midplane during metaphase, sister chromatids migrate in opposite directions to create the conditions required for the formation of the daughter nuclei and daughter cells during anaphase and successive telophase. Beyond that of chromosomes, the spindle also guides the segregation of centrosomes, of the Golgi apparatus, and, in certain cells, of the mitochondria [101].

The accuracy of chromosome segregation is striking. In budding yeast, only one error occurs in about 100,000 divisions [102]. In mammalian cells, in particular in cultured cell lines, the accuracy is lower [103]. In any case, the achievement is all the more remarkable when considering the scale: chromosomes are thousands of times larger than individual cytoskeletal motors, and mitotic movements span distances far exceeding the size of the molecular machines involved. The spindle must therefore orchestrate minute nanocomponents to move and partition massive structures with strict fidelity [99].

During telophase, chromatids tend to decompactify and be reorganized to ensure correct chromatin distribution, which includes the peripheral organization of heterochromatin and the redistribution of euchromatin into territories which will determine the transcriptional and epigenetic profiles of single cells [104,105]. Interestingly, in human cells, in anaphase and telophase, chromosome telomeres are found close to lamina-associated proteins [106,107,108]. This is believed to guide—at least partly—interphase chromatin organization [109,110].

In the final stages of mitosis, the spindle microtubules must be disassembled to permit the full reformation of the nuclear envelope. On the other hand, at the start of open mitosis, the nuclear envelope must be disassembled [90].

**Disassembly and reassembly of the nuclear envelope in mitosis**

In eukaryotic cells, the nuclear envelope separating the nuclear DNA from the cytoplasm is composed of an inner and an outer nuclear envelope. Beneath the nuclear envelope inner membrane resides the nuclear lamina, which plays an important role in the mechano-stability of the nucleus. The nuclear lamina is composed of nuclear lamins. The principal lamins are A-type and B-type and they are structurally organized in microfilaments of 3–5 nm diameter [111,112,113]. The nuclear envelope and lamin-associated proteins include LAP1 [114], LAP2/emerin/MAN1 (LEM)-domain proteins including LAP2β [115], emerin [116], MAN1 [117], LEM2 [118] and ANKLE2 [119], lamin B receptor (LBR) [120] and Sad1p/UNC-84 (SUN) domain proteins SUN1/2 [121,122].

The disassembly of the nuclear envelope in open mitosis begins with the release of nucleoporins, the constituents of nuclear pore complexes [123]. Successively, the tearing of the nuclear envelope follows, which is helped by the microtubules residing out of the nucleus and the disassembly is completed by the depolymerization of the nuclear lamina underlying the inner nuclear envelope of the nuclear envelope. During prophase, A-type lamins are released in the nucleoplasm, B-type lamins are reorganized, and nuclear envelope proteins are redistributed in the endoplasmic reticulum [124,125]. 

Lamin release and nuclear envelope disassembly are controlled by phosphorylation. Indeed, lamins, nuclear envelope components, nuclear pore complex components, and nuclear envelope/chromatin linking factors, such as BAF1, are phosphorylated by multiple kinases including CDK1, PKC, VRK1, aurora kinases, PLK1, and NIMA-related kinases [123]. CDK1 and PKC, by phosphorylating serine residues, cause the disassembly of the lamin filaments [126,127].

In the final stages of mitosis, the reverse process happens, i.e., nuclear envelope reassembly. During anaphase and telophase, while the chromatids slide along microtubules with the help of kinetochore motors to opposite poles, the nuclear envelope reassembles in the daughter nuclei. During this process, the multiple factors associated and composing the nuclear envelope wrap the decondensing chromatids. These include lamin A/C and lamin-associated proteins such as LAP2α, LAP2 β, LEM2 and emerin, and the chromatin-binding factor BAF1, lamin B, lamin B receptor and the components of the nuclear pore complexes [107,128,129,130,131,132,133,134]. How the recruitment of these factors and the completion of nuclear envelope assembly are intermingled with the ESCRT machinery will be discussed in the successive section.

As for nuclear envelope disassembly, the level of phosphorylation of nuclear envelope components controls nuclear envelope reassembly. Dephosphorylation is operated by the cell cycle-controlled PP1 and PP2A [135]. The process also involves the factor Repo-man which brings PP1 onto anaphase chromosomes [136]. 

## 4. The Roles of the ESCRT Machinery in Mitosis

In open mitosis, the steps involving the ESCRT machinery have been studied in depth. According to the current literature, canonical ESCRTs type I and II have not yet been identified at the nuclear envelope, while ESCRTs type III are typically recruited.

During prophase, the ESCRT type III, CHMP4C, has been shown to localize at kinetochores; its presence is then reduced at kinetochores aligned on the metaphase plate [137,138]. CHMP4C plays a role in the mitotic spindle checkpoint, independently of its membrane-related functions during cytokinesis [138]. CHMP4C is indeed required for the stable attachment of microtubules to kinetochores through the recruitment of the NDC80 complex proteins Hec1 and Nuf2, thereby ensuring proper chromosome alignment and segregation [137,138]. Despite its mitotic function, CHMP4C does not participate in nuclear envelope or membrane reassembly during the temporal window from anaphase to late telophase [139].

**Figure 3:**
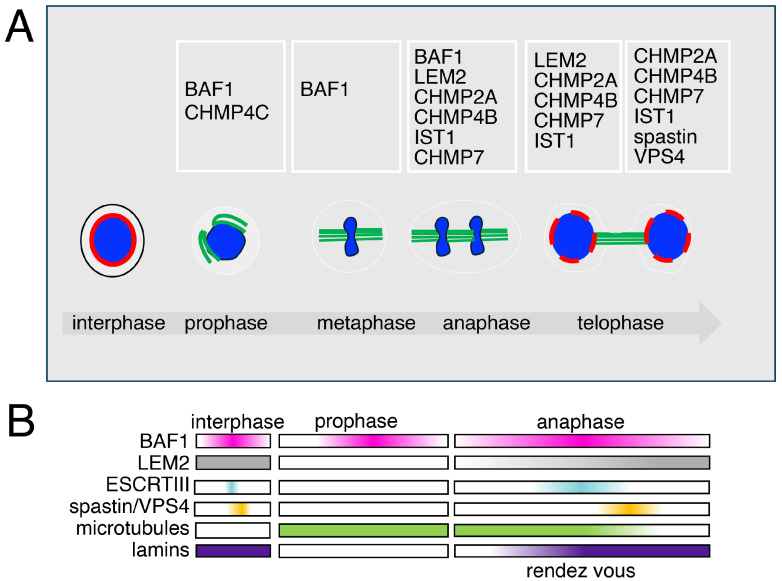
The different roles and dynamics of the ESCRT machinery in mitosis (**A**) Open mitosis is characterized in eukaryotes by the disassembly of the nuclear envelope components. BAF1 is found at spindle poles when cells enter mitosis, preceding the recruitment of the type III ESCRTs. CHMP4C, an ESCRT type III protein, is found at the kinetochores in prophase. During ana-telophase, ESCRTs type III are recruited at the chromatin core by BAF1 and LEM2 to promote nuclear envelope sealing. CHMP7, CHMP4B, CHMP2A, and IST1 coordinate the activity of VPS4 and Spastin, ensuring both ESCRT disassembly and spindle microtubule clearance. This dual action allows proper nuclear reformation and the reassembly of lamins and chromatin reorganization, a mechanism conserved from yeast to mammalian cells. (**B**) The dynamics of the recruitment of the chromatin core-associated factors.

From anaphase to telophase, a specific region at chromatin is involved in ESCRT aggregation and nuclear envelope reformation. This region, named core, is placed on the sides of anaphase chromatin and close to spindle microtubules. The core region is subdivided into each anaphase chromatin disk in inner and outer core regions—the inner core faces the mid-spindle and the outer core faces the spindle poles (Figure 3) [3,4,128,140]. 

The early literature, mainly based on cytological observations, including time lapse, FRAP and FRET analyses, defined the presence of an immobile aggregate at the core of chromatin in ana-telophase [140]. These studies also integrated with electron microscopy showed the presence in this aggregate of BAF1 and of nuclear envelope-associated proteins. The aggregate was also defined to be in close association with the microtubule spindle fibers [140]. These studies have now been integrated with those that define a BAF1-LEM2-ESCRT connection and give a phase separation mechanistic interpretation for the formation of the aggregate. Indeed, the recruitment, or rendez vous, of factors at the core has been shown to be coordinated by the non-ESCRT factors BAF1 and LEM2. To mechanistically explain the rendezvous, the main hypothesis based on the phase separation process, is enchained by the presence of BAF1 and LEM2, which would promote the recruitment of ESCRTs type III including CHMP2A, CHMP4B, CHMP7, and IST1 (CHMP8) [65,129,141]. 

As at other ESCRT-controlled sites, the ESCRTs type III working at the nuclear envelope recruit downstream ATPases. At the nuclear envelope, these are VPS4, regulating the disassembly of ESCRT polymers, and Spastin, an ATPase operating for the disassembly of microtubules. According to biochemical and genetic studies, VPS4 would be directly recruited by the ESCRT CHMP7, while IST1 (CHMP8) would be responsible for the recruitment of Spastin. In the absence of Spastin, spindle microtubules are not disassembled, impairing the correct finalization of mitosis, including nuclear envelope resealing and chromatin reorganization. In such conditions, DNA-damaged foci and an overall defective phenotype characterize the cell [4]. 

In sum, the generally accepted mechanistic scheme foresees that the ESCRT machinery at the nuclear envelope has, mitotically, a double function—that of repairing the nuclear envelope to avoid its along with that of contributing to the elimination of microtubule spindle fibers which hinder nuclear envelope reassembly. 

While the structural organization and dynamics of the ESCRT III biochemical complexes in anaphase and telophase have been thoroughly characterized and it has been defined that most factors but CHMP7 function as a partial replica of the same complex operating at other cell sites, the earlier moments of the assembly of the machinery during nuclear envelope reformation are different and less well-defined. In fact, the absence of ESCRTs type I and II in this process appears as an exception for the ESCRT organization in higher organisms. Moreover, if we consider an extension of the time window for the analysis of the ESCRT dynamics at the nuclear envelope, some poorly explored elements emerge. Namely, a more defined understanding of the phase preceding the rendezvous of ESCRT factors at anaphase core chromatin.

Following this line of reasoning, it is interesting to take into consideration a recent ESCRT study in which the authors have extended the analysis of the role of the ESCRT machinery taking a view that bypasses that of the anaphase and telophase stages. The work focuses on analyses performed in the fission yeast *Schizosaccharomyces japonicus*. The study re-links together the ana-telophase with interphase and takes into consideration the reassembly of the nuclear envelope and also the reorganization of heterochromatin. The authors propose a model in which the chromatin-binding factor Nur1 and the lamin-interacting protein Lem2 form a complex with heterochromatin [142]. Partly in analogy with what happens with LEM and BAF1 in human cells, the Lem2-Nur1 complex favors the local concentration of the yeast ortholog of CHMP7, Cmp7. Again, as in mammalian cells, Cmp7 recruits the ATPase Vps4 (ortholog of mammalian VPS4) which regulates the release of Cmp7 from Lem2 and presumably, that of Lem2 from heterochromatin. In other words, Cmp7 and VPS4 regulate the attachment of heterochromatin to the nuclear envelope via Lem2, an interaction which must be restored systematically at the end of mitosis to ensure the correct reorganization of (hetero)chromatin during interphase. Consistently, in Cmp7 depleted cells, the Lem2-Nur1 machinery remains associated with heterochromatin, compromising the correct reestablishment of post-mitotic nuclear organization and compartmentalization [3,4,142]. 

Intriguingly, a factor, named AKTIP, associated with ESCRTs type I, has been identified for its enrichment at the nuclear envelope of interphase nuclei [143,144]. Although it has not yet been described in nuclear envelope reassembly, this protein shares similarity with the ESCRT type I TSG101 and its reduction impacts on the recruitment of ESCRTs type III at the midbody during abscission [145]. The properties of AKTIP induce to hypothesize the possibility that new factors are still to be identified which could contribute to the nuclear reformation, and that redundancy of mechanisms can be foreseen for this activity. Interestingly, AKTIP possesses a further characteristic which connects it not only with the ESCRT machinery but also with heterochromatin. Indeed, AKTIP depletion generates a significant alteration of telomeres, which represent a particularly sensitive heterochromatic portion of the human genome [146].

Another aspect, that is not the object of this review, yet is important, is the control by the ESCRT machinery operating at the nuclear envelope on defective nuclear pore complexes [147]. In yeast, the assembly and surveillance of nuclear pore complexes is operated by an LEM factor, Heh2, which recruits ESCRTs type III [148]. The latter in turn recruit the Vps4 ATPase which contributes to the regulation and clearance of defective nuclear pore complexes [148,149,150].

Another pivotal activity of the ESCRT machinery is linked with the mechanism of repair of lysosome membranes [151,152]. ESCRTs type I, II, and III are involved in the repair of the damaged lysosomes: the process is started by the recruitment of TSG101 and ALIX, which then cooperate in the recruitment of the ESCRTs type III and of VPS4A [151]. Recently, experimental evidence has also shown that lysosomes play important regulatory roles in cell metabolism and during cell division [153,154]. Indeed, in mitosis, although cells can block or reduce the activity of processes not involved in cell division (i.e., transcription, translation, and autophagy), those involving lysosomes remain active [155]. Intriguingly, blocking lysosomal leakage at mitotic entry causes telomeric segregation defects, whereas telomere deprotection can trigger lysosome leakage [156]. Moreover, ESCRTs type III have been linked to vesicle-mediated nucleocytoplasmic transport. Finally, beyond their roles discussed so far, ESCRTs type III can act at the nuclear membrane during the nuclear egress of the Herpes Simplex Virus 1 (HSV-1) [157]. 

## 5. The ESCRT Activity at Nuclear Envelope Ruptures

A role for the ESCRT subunits at the nuclear envelope has been defined beyond their implication in mitosis. This role is exerted at nuclear envelope ruptures that can be generated by mechanical stress induced on the interphase cell and to its nucleus by internal or external forces [158,159]. The consequences of nuclear envelope ruptures are detrimental to cell viability because of two main reasons: the exposure of nuclear DNA to cytoplasmic factors, and epigenetic alterations due to the disorganization of the nuclear structure [160].

When, upon rupture, genomic DNA is exposed to the cytoplasm, DNA damage can occur as a consequence of the activity of cytoplasmic exonucleases, for example, TREX1 [161,162]. Damaged DNA enchains a response in the cell, in which ATM and ATR proteins are recruited at the site of damage and the sensor cGAS binds to cytoplasm-exposed DNA [163]. cGAS in turn activates the immune signaling cascade controlled by STING [164,165]. The activation of such a cellular response on the one side creates an overall alteration of the biology of the cell and, on the other hand, shows the importance for the cell to be able to repair ruptures and limit genomic DNA vulnerability [166].

For the second aspect, i.e., the epigenetic alterations, these have been analyzed in depth in lamin mutants which display fragile nuclei. The lamin D50 progeroid dominant mutation, for example, has been linked with alterations of epigenetic marks generated by nuclear envelope alterations [166,167,168,169].

In the process of nuclear rupture/alteration repair, the ESCRT subunits act, in part, as in post-mitotic nuclear envelope reassembly. The main route identified for this process is based on the recruitment of BAF1 on double-stranded DNA which is exposed to the cytoplasm [170,171]. Upon BAF1 accumulation at the site of rupture, cGAS is moved away to make room for DNA repair enzymes such as PARP1 [172,173]. For nuclear envelope repair, lamins and lamin-associated factors are recruited at the site of damage to create a plaque. This structure is a partial replica of the macromolecular aggregate condensating at the core of anaphase chromatin. Indeed, lamins, LAP2α and β, emerin, MAN1, LEM2 and ANKLE2 compose the plaque [171,174]. It is at this plaque that the ESCRT type III and the ATPase VPS4 are recruited [158]. Here, again, according to a scheme partly replicating the mitotic one, the ESCRT III subunits control the process, including CHMP7, CHMP4B, CHMP2A, and CHMP3 [4,158,159]. As post-mitotically, the BAF1-LEM2-CHMP7 interaction plays a critical role in interphase nuclear envelope repair [171,174]. Differently from what happens during mitotic nuclear envelope reassembly, the nuclear pore complex components are not recruited ruptured nuclear envelope sites of interphase nuclei [174,175].

## 6. The Implication of ESCRTs in Disease

Being involved in so many cellular processes, it is not surprising that the ESCRT factors have been associated with multiple pathological conditions, from cancer to neurological disease, from viral infection to cataract (Table 2).

The correlation with cancer can be assigned to the role of the ESCRT machinery in cell division. Indeed, a defect in this process generates genome instability, including aneuploidy and single-chromosome aberrations [210]. Several studies have highlighted the implication of the ESCRT type I TSG101 in cancer [177]. Namely, TSG101 has been linked with hepatocellular carcinoma, with ovarian cancer [176] and, in Drosophila, a connection has been suggested between TSG101 and neoplasia [29]. Interestingly, the TSG101-like factor AKTIP acts as a co-driver of cancer aggressiveness when tested in p53 knockout, cancer-modeling mice [211]. The ESCRTs type III CHMP3 and CHMP1A have also been associated with cancer. A CHMP4C-associated polymorphism (rs35094336, CHMP4C^T232^) was identified in humans and associated with defective abscission, DNA damage, genome instability, and loss of p53 [139].

Beyond cancer, a conspicuous body of literature links defects in ESCRT factors with alterations of neuronal development and with neurodegenerative diseases [212]. Drosophila model systems of Huntington disease, Amyotrophic lateral sclerosis, and of Frontotemporal lobar degeneration have highlighted the putative role of ESCRTs along with that of the ESCRT-associated ATPase VPS4 in neurodegeneration [72,194].

Association of ESCRT factors with neurodegeneration has also been established in mammalian—human, mouse, rat and monkey—cells. Among the ESCRT-controlled processes, autophagy and endosomal trafficking were frequently indicated as the mechanisms associating ESCRT defects with neurodegeneration [213]. ESCRT type I TSG101 and ESCRTs type III CHMP3 and CHMP2B were, for example, associated with defective removal of Huntingtin-positive aggregates [193].

The role of the ESCRT machinery at the nuclear envelope has been discovered later in time as compared to its role in trafficking and in abscission. As a consequence, the pathological implications of the ESCRT machinery, particularly in the context of nuclear envelope reformation, have been less extensively explored. However, a link has been established between the Hutchinson–Gilford progeria syndrome, a nuclear envelope-associated disease, and the ESCRT machinery [157]. It can be foreseen that a larger body of evidence will link in the future nuclear envelopathies with ESCRT factors. Importantly a connection has been established between nuclear envelope defects, ESCRTs and cancer invasion. Indeed, it has been suggested that the fragility of the nuclear envelope could contribute to the mechanical properties of cells when these are under the pressure of narrow spaces as it happens in tumor metastasis [158,159]. 

One final aspect relating the nuclear envelope role of the ESCRT machinery pertains the regulation of the nuclear pore complexes [214]. Abundant evidence links neurodegenerative disease to defects of nuclear pore complexes caused by mutation in their single components, the nucleoporins [214]. Amyotrophic lateral sclerosis has been, for example, associated with mutations of NUP50 [215], while ataxia and microcephaly have been linked with mutations of TPR [216]. Interestingly, it has been demonstrated that the ESCRT CHMP7 contributes to the accumulation and compartmentalization of defective nuclear pore complexes at the nuclear envelope in a dedicated structure named SINC [62]. Taken together, these data indicate that since the ESCRT machinery is needed to prevent the detrimental effects of altered nuclear pore complexes, ESCRTs subunits are expected be, on the one hand, also directly responsible for damage and, on the other hand, able to represent a route for new therapeutic strategies in related pathological conditions. In general terms, the latter concept could be extended to other ESCRT-based mechanisms and to the relative disease conditions. One relevant example is the usage of a peptide targeting the α-synuclein-ESCRT interaction to treat Parkinson neurodegeneration [217].

In sum, ESCRTs are conserved through evolution, play multiple and critical roles in the cell and in the organism, and represent a new druggable route for human diseases. These aspects taken together make this field of study of particularly large and deep significance.

## 7. Simple Summary

The ESCRT machinery is a conserved complex involved in endosomal trafficking and membrane remodeling. Recent evidence shows that it also regulates key steps of open mitosis, including nuclear envelope reformation and spindle disassembly. Its proper ac-tivity ensures mitotic fidelity, while its dysfunction causes genomic instability and con-tributes to diseases such as cancer and neurodegeneration. This review summarizes recent insights into how ESCRT components coordinate mitotic membrane dynamics under physiological and pathological conditions.

## Figures and Tables

**Table 2:** ESCRT components and diseases. ESCRT factors have been implicated in multiple pathological conditions, the most relevant of which are reported in the table. For each disease, the ESCRT factors involved, the specific alterations associated with the pathological condition, and the corresponding dysregulated pathway are indicated. ↑ increased; ↓ decreased; - wild type protein involved.

Disease	ESCRT Protein	Alteration	Affected Processes	Refs
ovarian cancer	TSG101	↑ expression	altered differentiation, proliferation, migration and apoptosis through MAPK signaling	[176,177]
breast cancer				[178]
thyroid cancer			altered equilibrium p53/MDM2	[179]
lung cancer			altered proliferation	[180]
hepatocellular carcinoma (HCC)	VPS37A	↓ expression	altered cell proliferation and invasion	[181]
prostate cancer	CHMP3	altered interactions	altered cell differentiation through interaction with IFG-binding protein family	[182]
lung cancer				[183]
hepatocellular carcinoma (HCC)		↑ expression	altered caspase-1-mediated pyroptosis pathway	[184]
pancreatic cancer	CHMP1A	↓ expression	Altered cell proliferation through p53 pathway	[185]
renal cell carcinoma (RCC)				[186]
ovarian cancer	CHMP4C	polymorphism rs35094336	altered cellular division checkpoint	[139]
prostate cancer				
skin cancer				
cervical carcinoma		↑ expression	altered cell proliferation and migration	[187]
lung cancer				[188]
prostate cancer				[189]
breast cancer	VPS4 A,B	↑ expression	altered cell proliferation	[190,191,192]
pancreatic cancer				
neurodegeneration (Huntington Disease (HD); Amyotrophic lateral sclerosis (ALS); Alzheimer’s Disease)	TSG101	lost from ER	altered endosomal and lysosomal sorting, affecting neuronal survival and clearance of protein aggregates	[193]
spongiform neurodegeneration	TSG101	interaction with Mahogunin	altered endosomal trafficking	[194]
Charcot–Marie–Tooth disease type 1C	TSG101	interaction with SIMPLE	altered lysosomal sorting	[195]
neurodevelopmental diseases	VPS22	mutations	altered autophagy	[196,197]
amyotrophic lateral sclerosis (ALS)	CHMP2B	mutations	dysfunction in endosomal–lysosomal sorting and autophagy	[196]
frontotemporal dementia (FTD)				[198]
hereditary spastic paraplegia (HSP)	CHMP3	mutations	altered autophagy	[199]
pontocerebellar hypoplasia (PCH)	CHMP1A	mutations	altered vesicles trafficking	[200,201,202]
C9orf72 amyotrophic lateral sclerosis/frontotemporal dementia (ALS/FTD) and sporadic ALS	CHMP7	↑ nuclear localization	altered nuclear pore complex homeostasis	[203]
congenital dyserythropoietic anemia (CDA) and neurodevelopmental disorder	VPS4	mutations	cytokinesis and trafficking defects	[204,205]
non-alcoholic fatty liver disease (NAFLD) and diabetes	VPS37A	↓ expression	altered endosomal signaling through Gcgr	[206]
hereditary cataracts	CHMP4B	mutations	altered endosomal sorting and altered autophagolysosomal degradation of micronuclei	[207,208]
many enveloped DNA or RNA virus infection	TSG101	-	involved in viral egress, viral budding, virion production, transport, RNA replication	reviewed in [209]
	VPS28	-	viral replication, transport and release	
	VPS25	-	viral replication, transport and release	
	VPS22	-	viral egress	
	VPS36	-	viral egress and budding	
	CHMP1A	-	viral trafficking	
	CHMP2A	-	viral budding	
	CHMP4A	-	viral replication and virion assembly	
	CHMP4C	-	Envelopment	
	CHMP4B/C	-	viral replication and release	
	VPS4A/B	-	entry, transport, viral egress, budding, replication and release	
